# Trade-Offs Among Aboveground, Belowground, and Soil Organic Carbon Stocks Along Altitudinal Gradients in Andean Tropical Montane Forests

**DOI:** 10.3389/fpls.2020.00106

**Published:** 2020-03-03

**Authors:** Lydia de la Cruz-Amo, Guillermo Bañares-de-Dios, Victoria Cala, Íñigo Granzow-de la Cerda, Carlos I. Espinosa, Alicia Ledo, Norma Salinas, Manuel J. Macía, Luis Cayuela

**Affiliations:** ^1^ Departamento de Biología y Geología, Física y Química Inorgánica, Universidad Rey Juan Carlos, Madrid, Spain; ^2^ Departamento de Geología y Geoquímica, Universidad Autónoma de Madrid, Madrid, Spain; ^3^ Instituto de Ecología, Universidad Técnica Particular de Loja, Loja, Ecuador; ^4^ Institute of Biological and Environmental Sciences, University of Aberdeen, Aberdeen, United Kingdom; ^5^ Instituto de Ciencias de la Naturaleza, Territorio y Energías Renovables, Pontificia Universidad Católica del Perú, Lima, Peru; ^6^ Departamento de Biología, Área de Botánica, Universidad Autónoma de Madrid, Madrid, Spain; ^7^ Centro de Investigación en Biodiversidad y Cambio Global (CIBC-UAM), Universidad Autónoma de Madrid, Madrid, Spain

**Keywords:** aboveground biomass, allometric equations, Andes, belowground biomass, climatic gradients, precipitation seasonality, soil organic carbon

## Abstract

Tropical montane forests (TMFs) play an important role as a carbon reservoir at a global scale. However, there is a lack of a comprehensive understanding on the variation in carbon storage across TMF compartments [namely aboveground biomass (AGB), belowground biomass (BGB), and soil organic matter] along altitudinal and environmental gradients and their potential trade-offs. This study aims to: 1) understand how carbon stocks vary along altitudinal gradients in Andean TMFs, and; 2) determine the influence of climate, particularly precipitation seasonality, on the distribution of carbon stocks across different forest compartments. The study was conducted in sixty 0.1 ha plots along two altitudinal gradients at the Podocarpus National Park (Ecuador) and Río Abiseo National Park (Peru). At each plot, we calculated the amount of carbon in AGB (i.e. aboveground carbon stock, AGC), BGB (i.e. belowground carbon stock, BGC), and soil organic matter (i.e. soil organic carbon stock, SOC). The mean total carbon stock was 244.76 ± 80.38 Mg ha^–1^ and 211.51 ± 46.95 Mg ha^–1^ in the Ecuadorian and Peruvian plots, respectively. Although AGC, BGC, and SOC showed different partitioning patterns along the altitudinal gradient both in Ecuador and Peru, total carbon stock did not change with altitude in either site. The combination of annual mean temperature and precipitation seasonality explained differences in the observed patterns of carbon stocks across forest compartments between the two sites. This study suggests that the greater precipitation seasonality of colder, higher altitudes may promote faster turnover rates of organic matter and nutrients and, consequently, less accumulation of SOC but greater AGC and BGC, compared to those sites with lesser precipitation seasonality. Our results demonstrate the capacity of TMFs to store substantial amounts of carbon and suggest the existence of a trade-off in carbon stocks among forest compartments, which could be partly driven by differences in precipitation seasonality, especially under the colder temperatures of high altitudes.

## Introduction

Tropical forests are the most important terrestrial carbon sink (Pan et al., 2011). During the last century, however, both the increase in temperatures and the loss of tropical forest cover may have diminished their effectiveness in mitigating the effect of climate change (Gibbs et al., 2007; Saatchi et al., 2011; Liu et al., 2017; Mitchard, 2018). In this context, it is paramount to make accurate estimations of the carbon content of tropical forests. Because programs such as REDD+ (Reducing Emissions from Deforestation and Forest Degradation; http://theredddesk.org/) are intended to provide economic rewards for developing countries that reduce their carbon emissions (Saatchi et al., 2011; Sills et al., 2017), knowing the amount of carbon stored in ecosystems is also key in political and economic terms—not just ecological—when designing effective policies.

Tropical montane forests (TMFs) in the Andes range across broad gradients, both altitudinal (typically from *ca*. 1000 to over 3600 m; Spracklen and Righelato, 2014) and environmental, making them unique for understanding the influence of climate on carbon stocks (Malhi et al., 2010). They are important by providing ecosystem services and as biodiversity hotspots (Bruijnzeel et al., 2011), but in comparison to moist lowland tropical forests, their role as a carbon sink is still poorly understood (e.g. Baker et al., 2004; Gibbs et al., 2007; Malhi et al., 2009; Malhi et al., 2010; Paulick et al., 2017). So far we know that TMFs can store substantially more aboveground biomass (AGB) per unit area than previously believed (global AGB average of 271 Mg ha^–1^), although their contribution to AGB per unit area is lesser than that of lowland tropical forests (global AGB average of 423 Mg ha^–1^; Spracklen and Righelato, 2014). Alternatively, TMFs could be important for the storage of carbon as belowground biomass (BGB, Leuschner et al., 2007; Girardin et al., 2010) and as soil organic matter (Raich et al., 2006; Leuschner et al., 2007; Leuschner et al., 2013). This fact can be particularly relevant at higher altitudes, where hydromorphic processes (i.e. water saturation in the soil associated under conditions of reduction) can prevail over podsolisation processes (i.e. downward migration of aluminium, iron and organic matter, and their accumulation in lower layers), thus resulting in lower rates of organic matter decomposition (Schawe et al., 2007) and larger concentration of soil organic carbon (SOC).

In contrast to SOC, aboveground (AGC) and belowground carbon (BGC) are expected to decline with altitude (Kitayama and Aiba, 2002; Raich et al., 2006; Girardin et al., 2010; Girardin et al., 2013; Phillips et al., 2019). However, other patterns have been also reported for the relationship between AGC and altitude: positive monotonic (Tashi et al., 2016), unimodal (Weaver and Murphy, 1990; Lieberman et al., 1996; Raich et al., 1997; Moser et al., 2008; Alves et al., 2010; Larjavaara and Muller-Landau, 2012; Marshall et al., 2012; Ensslin et al., 2015; Phillips et al., 2019), bimodal (Venter et al., 2017), or null (i.e. no relationship; Culmsee et al., 2010; Unger et al., 2012; Peña and Duque, 2013; Peña et al., 2018). There are fewer studies investigating changes in SOC along altitudinal gradients in TMFs. In addition to those reporting an increase of SOC with altitude (Townsend et al., 1995; Schrumpf et al., 2001; Kitayama and Aiba, 2002; Raich et al., 2006; Graefe et al., 2008; Girardin et al., 2010; Moser et al., 2011; Dieleman et al., 2013), some studies report no change at all (Soethe et al., 2007; Zimmermann et al., 2010; Phillips et al., 2019). Few studies have quantified carbon stocks in different TMF compartments (AGB, BGB, soil organic matter) simultaneously or total carbon stocks (Girardin et al., 2010; Phillips et al., 2019).

This study aims to fill some of the existing gaps of knowledge by investigating the role of Andean TMFs as carbon reservoirs. The specific goals were: (1) to understand how carbon stocks from the different forest compartments (AGB, BGB, and soil organic matter) and total carbon stock vary along the altitudinal gradient; and (2) to determine the influence of climate—particularly temperature and precipitation seasonality—on carbon stocks. Understanding these carbon trade-offs should be a priority when formulating policies and designing conservation and management plans aimed at mitigating consequences of environmental change (Mathez-Stiefel et al., 2017).

## Material and Methods

### Study Area and Climatic Characterization

The study was carried out along two altitudinal gradients of well preserved TMFs: Podocarpus National Park (Ecuador) and Río Abiseo National Park (Peru). These sites extend along wide altitudinal ranges (*ca.* 2000 m) of continuous forest cover, each within a single river basin: the Bombuscaro river in Ecuador and the Montecristo–Abiseo rivers in Peru. Three altitudinal belts were studied in each site: low (800–1,100 m), middle (1,900–2,100 m), and high (2,700–2,900 m). Sixty 0.1 ha (50 × 20 m) plots were established between 2015 and 2017: 10 plots within each belt at each site, at least 300 m apart (coordinates in **Appendix S1**) and avoiding natural disturbances (e.g., tree-fall gaps or landslides). A detailed description of the study area can be found in Bañares-de-Dios et al. (2020).

We retrieved bioclimatic variables from the CHELSA climatological dataset (Karger et al., 2017) for each gradient and selected two variables representing the main axis of climatic variability in our study sites: annual mean temperature (°C) and precipitation seasonality (%). The first was selected since TMFs extend along a broad thermal range (*ca*. 9 and 12 °C in Ecuador and Peru, respectively). Annual mean temperature was highly correlated with the rest of temperature-related bioclimatic variables (Pearson's correlation, r > 0.8). Precipitation seasonality was selected because it can have an important effect on soil mineralization rates and nutrient availability for plants, even though moist TMFs are not subjected to long periods of water deficit and thus its vegetation does not display adaptations to such conditions, like deciduousness. Precipitation seasonality was calculated as the standard deviation of the monthly precipitation estimates expressed as a percentage of the mean of those estimates (i.e. the annual mean). Thus, a higher value of precipitation seasonality means that the total monthly precipitation is more heterogeneously distributed across time. For instance, 50% of precipitation seasonality means that this variability represents 50% of the mean monthly precipitation. Because of the properties of the normal distribution, this implies that differences between the rainiest and driest months could be—with 95% confidence interval—as high as twice (i.e. 200%) the mean monthly precipitation value, whereas if precipitation seasonality were 10% this difference would be only of 40% of the mean monthly precipitation value.

### Field Sampling, Floristic, and Functional Data

In each plot, all standing woody stems (including trees, palms, tree ferns and lianas) ≥ 2.5 cm in diameter at breast height (DBH, at 130 cm from the ground) were recorded. For each stem, height was estimated visually since the use of precise instruments proved impractical. For individuals with multiple stems, we used the height of the tallest stem as the individual's height and the square root of the sum of the squares of DBH of each stem as the overall individual's DBH (Arellano et al., 2016). Field measurements were taken by multiple surveyors, who were trained at the beginning of each field campaign. Tree branch samples were collected for taxonomic determination and to estimate wood density. In total, 19,127 individuals were inventoried excluding tree ferns: 9,847 in Ecuador and 9,280 in Peru. Vouchers were identified at regional herbaria. In total, 826 different taxa were recognized from the Ecuadorian plots and 543 taxa from the Peruvian plots. The most abundant families in Ecuador were Melastomataceae (12.1% of total individuals), Lauraceae (10.8%), and Rubiaceae (9.0%) at low altitudes; Rubiaceae (16.5%), Melastomataceae (14.0%), and Lauraceae (12.7%) at mid altitudes; and Melastomataceae (21.5%), Cunoniaceae (16.8%), and Primulaceae (10%) at high altitudes. In Peru, the most abundant families were Leguminosae (8.9%), Rubiaceae (8.2%), and Malvaceae (8.2%) at low altitudes; Piperaceae (21.6%), Lauraceae (9.5%) and Rubiaceae (8.3%) at mid altitudes; and Rubiaceae (16%), Piperaceae (15.7%), and Chlorantaceae (14.6%) at high altitudes.

Branch wood density (WD) was measured based on Cornelissen et al. (2003) and used as a proxy for stem wood density, since both are strongly and positively correlated (Swenson and Enquist, 2008). Sections of branches (as cylindrical as possible) of *ca.* 10 cm long were stripped of cortex, and their volumes calculated as cylinders by measuring their diameter and length fresh. Branch wood density was calculated dividing fresh volume by dry mass after drying at 80°C for 48–72 h. Mean WD and its standard deviation (SD) were calculated for each species using sample values of all conspecifics. For species with just one sample, SD was estimated by multiplying WD by the SD/mean ratio averaged across all the samples in the entire dataset, where SD/mean ratio_Ecuador_ = 0.200 and SD/mean ratio_Peru_ = 0.234. Individuals for which WD values were lacking (e.g. emergent tall trees with inaccessible branches or lost samples) were assigned the mean WD of their family and their SD estimated as above. The latter included 611 individuals in 57 taxa from Ecuador (6.2% of the total individuals) and 735 in 59 taxa from Peru (8.5%). Finally, the 63 individuals from Ecuador (0.6% of the total individuals) and 117 from Peru (1.3% of the total individuals) with no WD measurement that could not be identified to family or belonged to families with no WD data (Cyclanthaceae, Dioscoreaceae, and Icacinaceae in Ecuador; Arecaceae and Humiriaceae in Peru), were removed from the analyses.

### Carbon Stocks

We used Chave's three-variable pantropical allometry model (Chave et al., 2014) to estimate aboveground biomass (AGB) for each individual tree:

$$AGB=0.0673\cdot {\left(WD\cdot DBH^{2}\cdot H\right)}^{0.976}$$

where *WD* wood density (g·cm^–3^), *DBH* diameter at breast height (cm), and *H* is height (m). We then calculated the plot AGB as the sum of the AGB of all trees within each plot. We used the R package “BIOMASS” (Réjou-Méchain et al., 2017) to calculate the uncertainty associated to the estimation of AGB at the tree level, both as a result of measurement error and intraspecific variability in the case of WD. For the estimation of height and DBH, we assumed that the error was normally distributed, with the SD being 5% of the estimated value, thus reflecting that larger trees were likely to produce larger errors in the estimation of height and DBH. For WD, the error followed a normal distribution parametrized by the mean WD and its SD. Then the measurement errors of H, WD and DBH were included into Chave's equation using a Bayesian inference procedure (1000 iterations; Chave et al., 2014). The AGB of lianas was calculated separately through the following equation (Schnitzer et al., 2006):

$$AGB=e^{-1.484+2.657\cdot ln\left(DBH\right)}$$

and we added it to each plot's estimated AGB.

Belowground biomass (BGB) was estimated using Kachamba et al. (2016)'s equation:

$$BGB=0.285\cdot DBH^{1.993}$$

We assumed that the aboveground carbon stock (AGC) of a plot accounted for the 50% of its AGB, following Chave et al. (2005). We replicated this assumption for belowground carbon stock (BGC) from BGB.

Soil samples were collected from the surface (0-15 cm) below the decomposing organic layer (e.g., foliage, small twigs, fruits, seeds) in each plot. Soil samples, consisting of five different subsamples from five different points in the plot, were collected, mixed, air-dried and sifted through a 2-mm sieve (Arellano et al., 2016). Soil pH was determined in 1:2.5 pH-deionized water, soil texture was determined by the Bouyoucos hydrometer method (Bouyoucos, 1962), and total C and N concentrations were measured through dry combustion using a LECO CHNS-932 elemental auto-analyzer. Bulk density was determined by core sampling at three points of each plot. Soil samples were dried at 105 °C for 24 h. Total oven-dry mass of the soil samples were weighted, and then the coarse fraction (>2mm) was separated and weighted to determine the gravimetric coarse fraction content. The plant residue (mainly coarse roots) included in the coarse fraction (>2 mm) was weighted separately in order to determine the percentage present in the whole sample. Bulk density (BD) was calculated as $Mt/\sum_{i=1}^{n}Vi$ where *Mt* is the total oven-dry mass of the three cores (g), and *Vi* the volume of each core *i* (cm^3^). Finally, the soil organic carbon stock (SOC) (Mg ha^–1^) was calculated following Schrumpf et al. (2011):

$$SOC=\frac{C\cdot BD\cdot LTH\cdot FEcontent}{10}$$

where *C* is soil organic carbon concentration (g kg^–1^) in the soil layer, *BD* bulk density (g cm^–3^), *LTH* layer thickness (cm), and *FE content* the relative contribution of fine earth fraction (i.e. all soil particles smaller than 2 mm, thus excluding gravel, stones and coarse plant residue) to total soil mass. Despite important variations in BD across sites and altitudinal ranges (see **Appendix S3**) that make seemingly reasonable to correct SOC estimates by an equivalent soil mass, SOC has been historically quantified to a fixed depth. This method has been employed in the vast majority of publications comparing SOC between treatments or over time periods (Wendt and Hauser, 2013), it is designated as good practice by the Intergovernmental Panel on Climate Change (IPCC, 2003), and has been subsequently used in protocols of global importance to assess SOC, such as that of the European Joint Research Centre (JRC-EU; Stolbovoy et al., 2007).

### Relation Between Carbon Stocks, Altitude, and Climate

Generalized linear models (GLMs) with a Gamma error distribution were fitted to determine how carbon stocks vary in the different compartments of the forest and along the altitudinal gradient separately for Ecuador and Peru. Quadratic terms of the explanatory variables were included to account for non-linear relationships. To understand the role of climate on the carbon stocks of the different compartments, we fitted another set of GLMs with a Gamma error distribution to relate each of the three carbon stock response variables, as well as total carbon stock, to annual mean temperature (°C) and precipitation seasonality, as well as their interaction, using data from both sites. The significance of predictors was tested using the Chi-squared statistic (α ≤ 0.05), and the explained deviance (*D* ^2^) was used to assess the goodness of fit of the model:

$$D^{2}=\frac{\left(nulldeviance-residualdeviance\right)}{nulldeviance}$$

All analyses were conducted with the R environment (R Development R Core Team, 2018).

## Results

### Climatic Characteristics and Soil Properties

Annual mean temperature and temperature seasonality decreased consistently as altitude increased at both sites. Temperature range was broader in the Peruvian (11.5–23.9 °C annual mean) than in the Ecuadorian site (12.2–21.1 °C annual mean) (**Appendix S2**). Patterns of annual precipitation differed between sites: while in the Ecuadorian site precipitation increased with altitude (from 957 to 1,614 mm/year), in the Peruvian site it ranged from 1,019 to 2,007 mm/year, and was lowest at the mid altitudinal belt (**Appendix S2**). The range of precipitation seasonality in Ecuador was much narrower (24.3–32.9%) than in Peru (31.8–50.8%), where it increased from low to middle and high altitudes (**Appendix S2**).

Soil pH ranged from 2.31 to 5.98 in Ecuador and from 3.64 to 6.83 in Peru. Soils from Ecuador showed consistently lower pH values and higher organic carbon concentration, C/N ratio, and bulk density than those from Peru at each altitudinal belt (**Appendix S3**). There was an overall decrease in pH and an increase in organic carbon concentration and C/N ratio with altitude (**Appendix S3**). Soils from Ecuadorian plots showed a similar sandy loam texture along the altitudinal gradient, while in the Peruvian plots soil texture ranged from loam, at low altitude, to sandy loam at middle altitude, and loamy sand at high altitude (**Appendix S3**). In both sites, bulk density gradually decreased with altitude. Soils from Ecuador showed consistently lower pH values and higher organic carbon concentration, C/N ratio, and bulk density than those from Peru at each altitudinal belt (**Appendix S3**).

### Carbon Stocks Along the Altitudinal Gradient

Mean diameter at breast height (DBH) and tree height increased in Ecuador from low to mid altitudes and then decreased again at high altitude, whereas in Peru these values did not change much across altitudinal belts (**Appendix S4**). Wood density remained mostly constant across altitudinal belts in Ecuador, with a slight average increase at higher altitude, whereas in Peru average wood density values decreased monotonically with altitude (**Appendix S4**).

**Figure 1:**
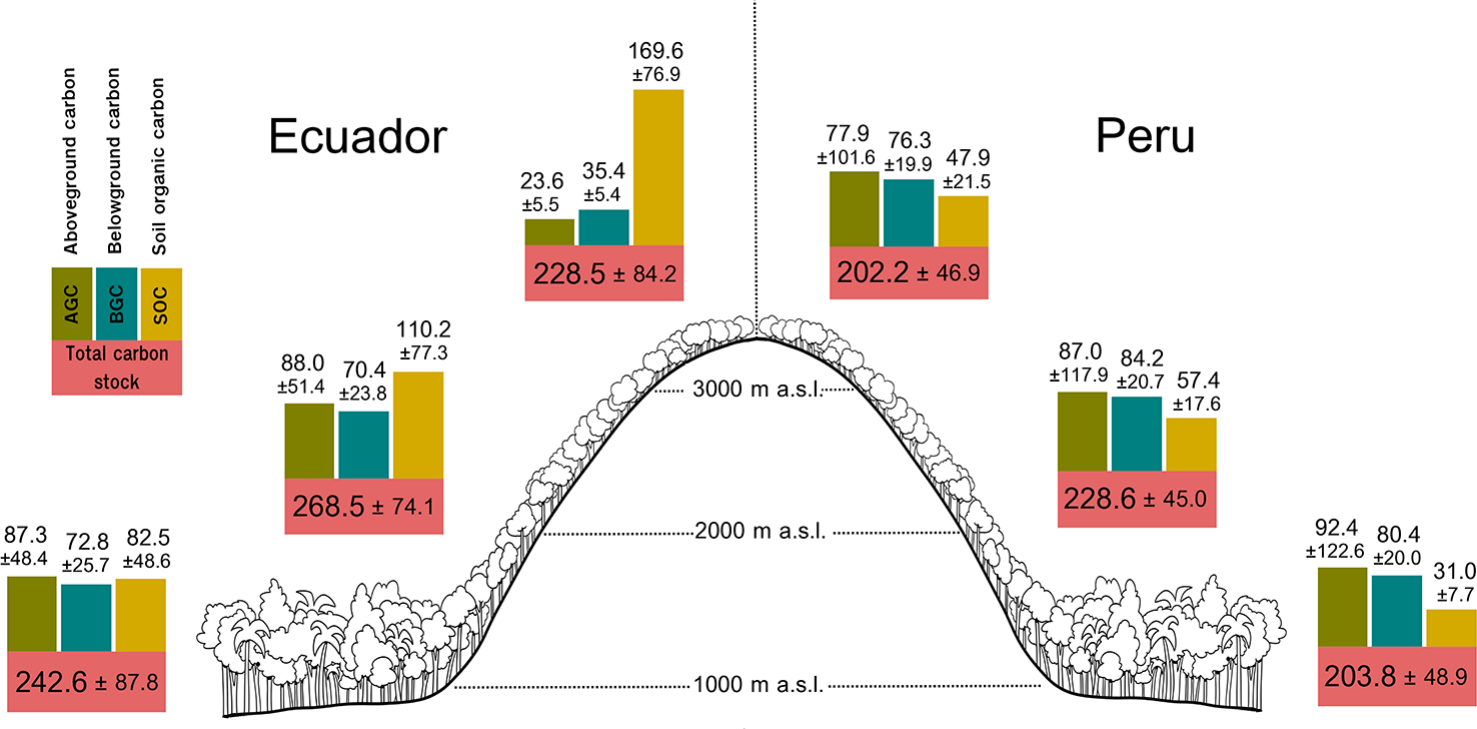
Carbon stocks (mean ± sd) for each forest compartment (above and belowground biomass, and soil organic carbon) and total carbon stock per altitudinal belt (drawing by María Medel).

AGC ranged from 16.82 to 222.07 Mg ha^–1^ among Ecuadorian plots, and from 37.14 to 160.17 Mg ha^–1^ among Peruvian plots (**Figure 1**). BGC ranged from 26.79 to 126.65 Mg ha^–1^ in Ecuador, and from 46.62 to 119.94 Mg ha^–1^ in Peru. SOC ranged from 26.65 to 268.09 Mg ha^–1^ in Ecuador, and from 12.87 to 100.38 Mg ha^–1^ in Peru. Total carbon stock ranged from 86.85 to 406.35 Mg ha^–1^ in Ecuador (mean value 244,76 ± 48.40 Mg ha^–1^), and from 116.73 to 318.52 Mg ha^–1^ in Peru (mean value 211.51 ± 80.38 Mg ha^–1^). The mean total carbon stock found across all study sites was 229.02 ± 68.06 Mg ha^–1^. All plot-level estimations of AGC, BGC, and SOC are available in **Appendix S1**.

**Figure 2:**
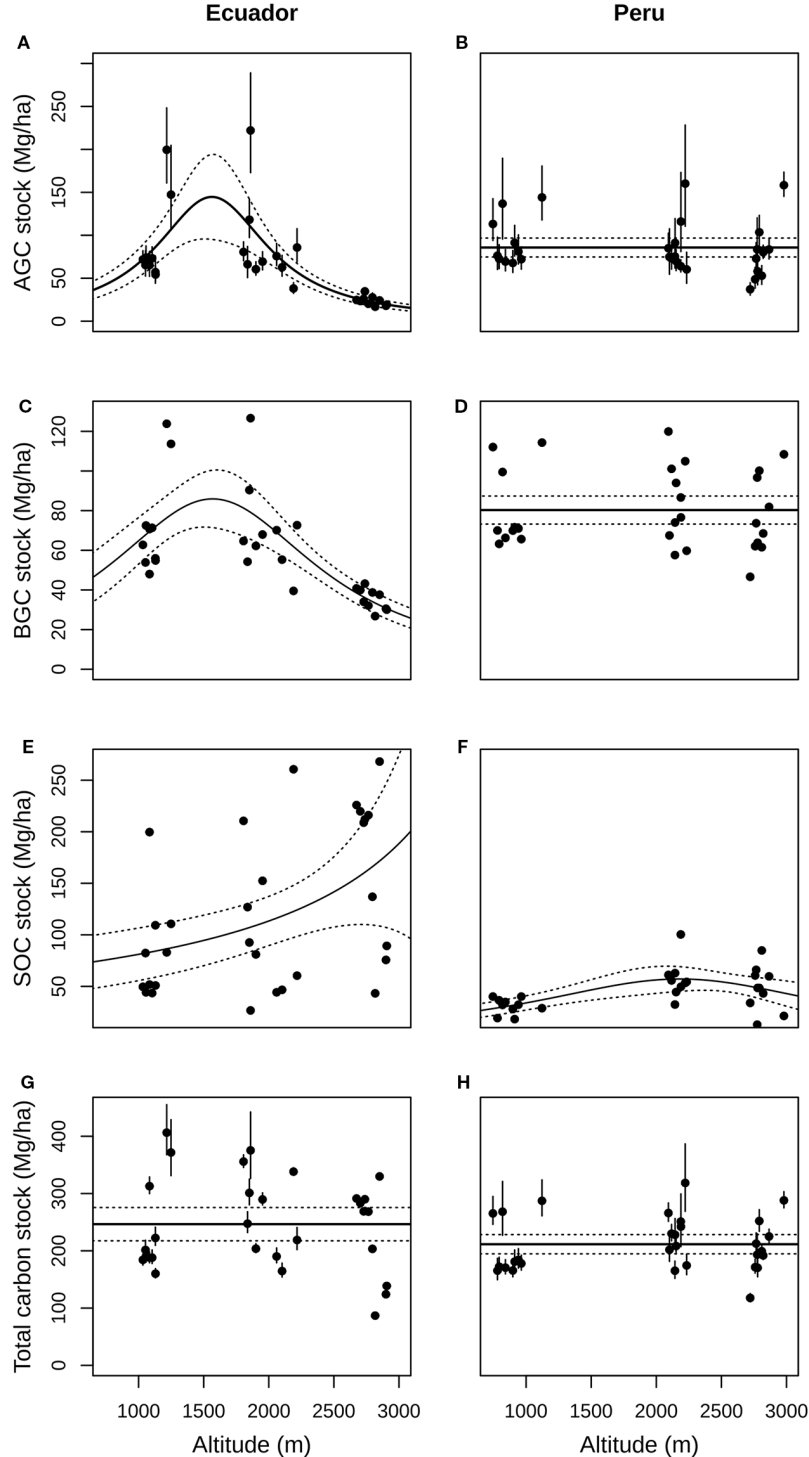
Relationship between aboveground **(A, B)**, belowground **(C, D)**, soil organic **(E, F)** and total carbon stocks **(G, H)** and altitude (m a.s.l), both in Ecuador (left charts) and Peru (right charts), with its 95% confidence intervals (dotted lines). AGC, Aboveground carbon; BGC, Belowground carbon; SOC, Soil organic carbon.

The largest mean AGC was found at low (87.34 ± 48.40 Mg ha^–1^) and middle altitudes (88.03 ± 51.39 Mg ha^–1^) from Ecuador, and decreased by *ca.* 73.2% at high altitude (23.56 ± 5.47 Mg ha^–1^; **Figure 1**); whereas in Peru, the greatest mean AGC was found at low altitude (92.44 ± 122.62 Mg ha^–1^), decreasing by *ca.* 6% at mid altitude (86.97 ± 117.86 Mg ha^–1^) and *ca.* 15.7% at high altitude (77.90 ± 101.56 Mg ha^–1^; **Figure 1**). In Ecuador, the best model for aboveground carbon (AGC) contained the quadratic term of altitude (**Table 1A**), and the predicted response curve revealed a unimodal relationship, reaching a maximum estimated AGC at *ca.* 1500 *m* (**Figure 2A**). In Peru, however, AGC did not show any relationship with altitude (**Figure 2B**, **Table 1A**).

**Table 1:** Analysis of deviance tables for the generalized linear models testing the effect of altitude (linear and quadratic terms) on different carbon stocks, namely aboveground carbon (AGC) in Mg/ha, belowground carbon (BGC) in Mg/ha, soil organic carbon (SOC) in Mg/ha, and total carbon stocks, in a) Ecuador and b) Peru, respectively.

**Response variable**	Term	**Deviance**	**d.f.**	**p-value**	**D^2^**	**Estimate**	**Std. Error**
a) Ecuador							
**AGC**	**Linear**	**4.700**	**1**	**<0.001**	**0.333**	**–7.757 10^–^^5^**	**1.356 10^–^^5^**
	**Quadratic**	**6.220**	**1**	**<0.001**	**0.441**	**2.481 10^–^^8^**	**4.014 10^–^^9^**
**BGC**	**Linear**	**1.947**	**1**	**<0.001**	**0.381**	**–3.683 10^–^^5^**	**8.902 10^–^^6^**
	**Quadratic**	**1.524**	**1**	**<0.001**	**0.298**	**1.174 10^–^^8^**	**2.436 10^–^^9^**
**SOC**	**Linear**	**2.400**	**1**	**0.008**	**0.192**	**–8.584 10^–^^6^**	**1.137 10^–^^5^**
	Quadratic	0.682	1	0.654	0.005	1.237 10^–9^	2.746 10^–9^
Total carbon stock	Linear	0.025	1	0.627	0.007	–4.980 10^–6^	2.711 10^–6^
	Quadratic	0.385	1	0.058	0.110	1.330 10^–9^	6.943 10^–10^
b) Peru							
AGC	Linear	0.075	1	0.462	0.022	1.472 10^–6^	7.106 10^–6^
	Quadratic	0.002	1	0.915	0.000	–2.117 10^–10^	1.977 10^–9^
BGC	Linear	0.002	1	0.852	0.001	–2.432 10^–6^	5.076 10^–6^
	Quadratic	0.017	1	0.611	0.010	7.167 10^–10^	1.405 10^–9^
**SOC**	**Linear**	**1.031**	**1**	**0.004**	**0.180**	**–3.739 10^–^^5^**	**1.222 10^–^^5^**
	**Quadratic**	**0.849**	**1**	**0.008**	**0.148**	**8.573 10^–^^9^**	**3.222 10^–^^9^**
Total carbon stock	Linear	0.007	1	0.717	0.005	–1.647 10^–6^	1.711 10^–6^
	Quadratic	0.043	1	0.360	0.030	4.334 10^–10^	4.716 10^–10^

Deviance, degrees of freedom (d.f.), p-values and explained deviance (D^2^) is shown for each term in the models. The estimated coefficients and their standard errors are also shown. Statistically significant terms (p-value ≤ 0.05) are highlighted in bold.

Regarding BGC, the largest mean value in Ecuador was found at low (72.75 ± 25.74 Mg ha^–1^); and middle altitudes (70.37 ± 23.85 Mg ha^–1^) and decreased by 49.7% at high altitude (35.38 ± 5.43 Mg ha^–1^; **Figure 1**); whereas in Peru, the largest mean BGC was found at middle altitude (84.21 ± 20.66 Mg ha^–1^; **Figure 1**). Overall, BGC followed the same relationship with altitude as AGC, both in Ecuador and Peru (**Figures 2C, D**, respectively; **Table 1**).

At each altitude, SOC in Ecuadorian plots were in general higher than in Peruvian plots. In Ecuador, the largest mean SOC was found at high (169.6 ± 76.9 Mg ha^–1^) and middle altitudes (1,10.2 ± 77.3 Mg ha^–1^) and the lowest mean SOC at low altitude (82.5 ± 48.6 Mg ha^–1^; **Figure 1**). Nevertheless, in Peru, the largest mean SOC was found at middle altitude (57.4 ± 17.6 Mg ha^–1^), though the differences across altitudinal belts were not as large as in Ecuador (**Figure 1**). SOC increased with altitude in Ecuador, while in Peru slightly increased from low to middle altitude and then stabilizing from middle to high altitude, with the relationship being quadratic (**Figures 2E, F**; **Table 1**).

The resulting total carbon stock (the sum of AGC, BGC and SOC) did not vary with altitude, neither in Ecuador nor in Peru (**Figures 2G, H**, respectively; **Table 1**).

### Carbon Stocks and Climate

**Figure 3:**
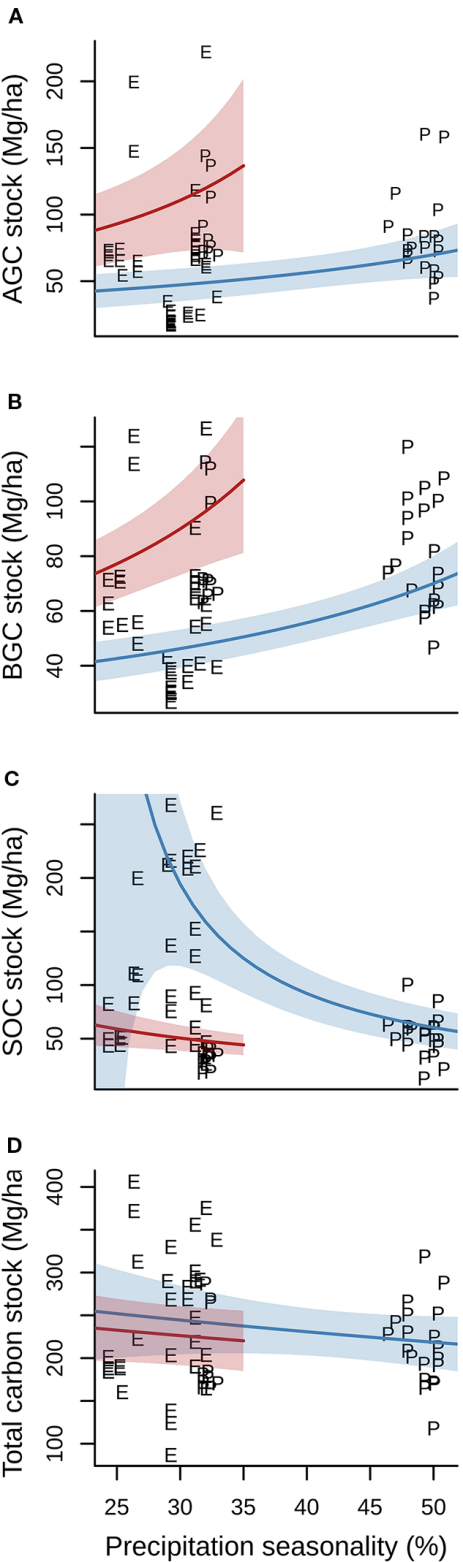
Relationship between aboveground **(A)**, belowground **(B)**, soil organic **(C)** and total carbon stocks **(D)** and precipitation seasonality (%) both under warm (24°C annual mean temperature, red lines) and cold (12°C, blue lines) conditions. E, Ecuadorian plots; P, Peruvian plots. Precipitation seasonality is calculated as the standard deviation of the monthly precipitation estimates expressed as a percentage of the mean of those estimates (i.e. the annual mean). The range of predictions for increasing values of precipitation seasonality (x-axis) represent the observed values in our two study sites, with lower variation at low altitude (i.e. warm conditions) and higher variation at high altitude (i.e. cold conditions). Shaded areas represent 95% confidence intervals upon model predictions.

Annual mean temperature and precipitation seasonality, but not their interaction, had a statistical significant effect on AGC, BGC, and SOC. There was, however, no effect of either climate variable or their interaction on total carbon stocks (**Table 2**). Precipitation seasonality had a positive effect on both AGC and BGC at low (i.e. warm temperatures, represented by red lines in **Figures 3A, B**) and at high altitudes (i.e. cold temperatures, represented by blue lines in **Figures 3A, B**). Temperature also had a positive effect on both AGC and BGC. Whereas the effect of precipitation seasonality on SOC was almost negligible when annual mean temperature was warm, there was a very marked negative effect when cold (**Figure 3C**). Finally, the total carbon stock did not show any difference with precipitation seasonality, either under warm or cold annual mean temperature (**Figure 3D**).

**Table 2:** Analysis of deviance tables for the generalized linear models testing the effect of annual mean temperature (T, in °C), precipitation seasonality (PS, in %), and their interaction (T:PS) on different carbon stocks, namely aboveground carbon (AGC), belowground carbon (BGC), soil organic carbon (SOC), and total carbon stocks. Deviance, degrees of freedom (d.f.), p-values and explained deviance (D^2^) is shown for each term in the models.

Response variable	Term	Deviance	d.f.	p-value	D^2^	Estimate	Std. Error
**AGC**	**T**	**1.921**	**1**	**0.017**	**0.104**	**–3.606 10^–^^4^**	**1.574 10^–^^4^**
	**PS**	**2.231**	**1**	**0.010**	**0.121**	**–1.524 10^–^^3^**	**7.103 10^–^^4^**
	T:PS	0.954	1	0.093	0.052	7.555 10^–6^	4.438 10^–3^
**BGC**	**T**	**0.728**	**1**	**0.008**	**0.089**	**–2.195 10^–^^4^**	**9.217 10^–^^5^**
	**PS**	**2.285**	**1**	**<0.001**	**0.280**	**–9.662 10^–^^4^**	**4.158 10^–^^4^**
	T:PS	0.219	1	0.145	0.207	3.884 10^–6^	2.644 10^–6^
**SOC**	**T**	**4.463**	**1**	**<0.001**	**0.139**	**2.037 10^–^^4^**	**1.659 10^–^^4^**
	**PS**	**9.283**	**1**	**<0.001**	**0.290**	**9.664 10^–^^4^**	**8.058 10^–^^4^**
	T:PS	0.087	1	0.615	0.003	–2.770 10^–6^	5.547 10^–6^
Total carbon stock	T	0.000	1	0.997	0.000	1.397 10^–5^	2.501 10^–5^
	PS	0.124	1	0.223	0.023	7.666 10^–5^	1.165 10^–4^
	T:PS	0.018	1	0.646	0.003	–3.549 10^–7^	7.765 10^–7^

The estimated coefficients and their standard errors are also shown. Statistically significant terms (p-value ≤ 0.05) are highlighted in bold. Precipitation seasonality is calculated as the standard deviation of the monthly precipitation estimates expressed as a percentage of the mean of those estimates (i.e. the annual mean).

## Discussion

Our results show that carbon storage at each compartment of the forest (AGC, BGC, and SOC) follows a distinct pattern along the altitudinal gradient that differs between sites.

Although AGC is expected to decline with altitude as a result of lower-statured trees (Kitayama and Aiba, 2002; Raich et al., 2006; Girardin et al., 2010; Girardin et al., 2013), in Ecuador our model shows maximum AGC at middle altitude (*ca.* 1500 m). This result is similar to the unimodal patterns reported in other studies (Weaver and Murphy, 1990; Lieberman et al., 1996; Raich et al., 1997; Moser et al., 2008; Alves et al., 2010; Larjavaara and Muller-Landau, 2012; Marshall et al., 2012; Ensslin et al., 2015; Phillips et al., 2019), and it has been suggested to be caused by a complex combination of factors that occur at mid altitudes, including optimal balance of respiration (with respiration costs being lower at mid altitudes) and photosynthesis (which is not yet inhibited by low air temperatures) and being less prone to disturbance compared to low and high altitudes (Marshall et al., 2012). Conversely, in Peru there was no relationship between AGC and altitude. No change of AGC with altitude has been also reported in other studies, whether caused by the dominance of Fagaceae that can reach very large sizes at high altitudes (Culmsee et al., 2010; Peña et al., 2018), or by increased soil fertility with altitude, which would have a positive effect on plant growth compared to poorer lowland soils, thus compensating the negative effect that lower temperatures have on AGC (Unger et al., 2012). The latter is more likely to explain the lack of an AGC pattern with altitude seen in Peru.

The same pattern of variation of AGC with altitude was found for BGC in both sites. This is not surprising as the equations for estimating AGC and BGC both relied on tree DBH. In general, coarse-root biomass contains the largest fraction of BGC, even if carbon stored in fine-root biomass can, in relative terms, be particularly high in upper TMFs (up to 46.0% of total BGC; Girardin et al., 2010), probably as a consequence of limited soil nutrients available (Leuschner et al., 2007; Girardin et al., 2010). Thus, it seems reasonable to assume that coarse root biomass, and therefore BGC, will be positively correlated with AGC because taller trees (more abundant at lower TMFs) would invest more into coarse-root anchoring—particularly in windy or erosion-prone sites—whereas smaller trees (more abundant at upper TMFs) will not be able to develop a large deep root system (Girardin et al., 2010).

In general, our results confirm that TMFs soil properties such as acidity, soil organic matter accumulation and C/N ratio increase with altitude, as reported in prior studies (Grieve et al., 1990; Schawe et al., 2007; Schrumpf et al., 2011). Yet, there were differences in the patterns of SOC along the altitudinal gradient between the two sites. Upper TMFs in Ecuador can store large amounts of SOC compared to forests at low altitudes, which is in agreement with patterns reported in previous studies for TMFs (Townsend et al., 1995; Schrumpf et al., 2001; Kitayama and Aiba, 2002; Raich et al., 2006; Graefe et al., 2008; Girardin et al., 2010; Moser et al., 2011; Dieleman et al., 2013). Contrastingly, in Peru, SOC slightly increased from low to middle altitude and remained unchanged above. Similarly, Schawe et al. (2007) observed in Bolivian TMFs a continuous increase of SOC up to 2000–2400 m that remained constant above that altitude. The increase in SOC at higher altitude in the Ecuadorian gradient might be partly explained by an increased C/N ratio as a consequence of low temperature and high precipitation that may cause soil acidification and subsequent low biological activity. However, this reasoning is not valid for explaining the lower SOC at the high altitude TMF in Peru. It is interesting to note that, on average, upper TMF soils in Peru showed higher organic carbon concentration (**Appendix S3**), but proportionally lesser bulk density, which ultimately explains the decrease in SOC from middle to high altitude. Typically, soils with higher organic content have lesser bulk density, although the bulk density of organic horizons also depends on the degree of decomposition, the make-up of plant residue in the soil and the void ratio. A greater amount of fiber or coarse roots in the core soil create a more open structure that results in more voids and therefore lower bulk density. The coarse-root (>2mm in diameter) fraction present in soils at high altitude in Peru constitute a much higher percentage of total soil weight than those from Ecuador, which may partly explain the lower value of bulk density of soils from Peru at high altitude, and will ultimately have a great influence on the calculation of the carbon stock of these soils.

Overall, regardless of the individual patterns of AGC, BGC, or SOC along the altitudinal gradient, we did not observe any variation with altitude at either study site when carbon stocks from all compartments were combined. This pattern has been also found by other studies (Raich et al., 2006; Girardin et al., 2010; Selmants et al., 2014; Phillips et al., 2019). It is likely the result of a trade-off among carbon stocks stored at different forest compartments. The key question, thus, is why different carbon stocks (AGC, BGC, and SOC) show diverging patterns of change with altitude between sites, but when combined they result in similar total carbon stocks that remain constant across altitude. It has been suggested that the lack of response to altitude of total carbon stocks is the result of changes in the prevailing atmospheric temperatures, which would trigger opposite trends in AGC and SOC, thus equalising total carbon stocks along the altitudinal gradient (Selmants et al., 2014; Phillips et al., 2019). However, this does not explain the disparity in the patterns of AGC, BGC and SOC reported in various studies of TMFs. We believe that there are other climatic drivers that may be playing an important role in determining the observed trade-offs, allowing for different patterns of change of the carbon stocks stored in the individual forest compartments—as reported in a number of studies—while maintaining total carbon stocks in a plot constant across altitude. Our analyses suggest that at low altitudes, warmer temperatures may promote nutrient cycling and plant growth (Kitayama and Aiba, 2002; Girardin et al., 2010) that contribute to high AGC and BGC, but also result in low SOC values. At those low altitudes, little variation in precipitation seasonality was observed between Ecuador (average value of 32.2%) and Peru (average value of 25.5%; **Appendix S2**). Higher precipitation seasonality therefore contributed to higher AGC and BGC, but showed little effect on SOC. Conversely, at high altitudes we found different patterns of precipitation seasonality: whereas our forest plots in the upper belt in Ecuador showed similar values of precipitation seasonality to those found at low altitudes (average value 29.7%), these were much higher for our plots in Peru (average value 50.2%; **Appendix S2**). This fact could explain the differences in the observed patterns of carbon stock among forest compartments between Ecuador and Peru. At high altitude in Ecuador, low precipitation seasonality results in 1) an accumulation of organic matter in the soil that renders high SOC values (Schawe et al., 2007) and, since available nutrients subsequently become scarce (due to low nutrient cycling), 2) a decrease in plant growth, as shown by the low AGC and BGC values. Conversely, the higher precipitation seasonality at greater altitude in Peru allows for drier periods during which mineralisation rates increase. The latter may result in 1) less accumulation of organic matter in the soil—as indicated by the decrease in SOC values—that allows for a richer pool of nutrients available to plants, and thus 2) favor an increase in plant growth, as shown by greater AGC and BGC values. Yet, we acknowledge that our conclusions should be taken with caution, as biotic drivers (e.g. soil food-web, plant competition aboveground, etc.) and variables influencing soil dynamics (e.g. soil type, plant residue quality and quantity, amount and quality of soil organic matter, microbial activity and composition) are also likely to contribute significantly to carbon stocks.

Ultimately, TMFs can make an important contribution to global carbon stocks. Our estimates of AGC (80.42 Mg ha^–1^ on average) are somewhat lower than those reported for other Andean TMFs (106.04 Mg ha^–1^ on average; Spracklen and Righelato, 2014). This could be partly a consequence of using branch wood density as a proxy for stem wood density, which could result in lower AGB and AGC estimated values, particularly in species with very hard wood species and/or large individuals, that could ultimately account for an important portion of a community's biomass. There is, however, enormous variation among studies, with estimates ranging from 77.20 Mg ha^–1^ (Girardin et al., 2010) to 409.07 Mg ha^–1^ (Grimm and Fassbender, 1981), proving the need for further studies. In general terms, although evergreen tropical lowland forests can store larger AGC than Andean TMFs (e.g. Baker et al., 2004; Malhi et al., 2009; Spracklen and Righelato, 2014), the latter—especially those at higher altitudes—can store a substantial amount of carbon in the form of SOC (Raich et al., 2006), a component of the system that has been often neglected.

The mean total carbon stock found in our study sites (229.02 ± 68.06 Mg ha^–1^) was within the low to middle range (87–754 Mg ha^–1^) reported in a meta-analysis of TMFs by Raich et al. (2006), and similar to the results recently reported for Colombian TMFs (241.3 ± 37.5 Mg ha^–1^; Phillips et al., 2019). Yet, according to our study, and considering that the extent of TMFs in the Neotropics has been recently estimated to be 750,000 km^2^ (Kappelle and Brown, 2001), New World TMFs could store 16–19 10^9^ Mg C, and up to 48–58 10^9^ Mg C, for the estimated *ca*. 2.2 10^6^ km^2^ of TMF worldwide (Mulligan, 2010). Although there is considerable uncertainty in these predictions—as is often the case when it comes to standing biomass estimation—they can be used to illustrate how large amounts of carbon in TMFs from both biomass and soils, are as much at risk of emission through deforestation and land use change as tropical lowland forests, with the consequent dramatic impact on global climate.

### Conclusions

Our study emphasizes the important role that TMF plays in storing carbon. It points out the existence of trade-offs among carbon stocks when partitioning the three TMFs compartments—AGB, BGB, and soil organic matter. The result is that plot overall carbon stock along the altitudinal gradient remains constant, although patterns of variation differed between sites when looking at individual components of the system. Precipitation seasonality may partly explain these differences, as the occurrence of drier periods in the year would increase mineralization rates, particularly at higher altitudes. This would result in less accumulation of organic matter in the soil and, consequently, an increase in plant growth and AGB. Such trade-offs reflect the potential of TMFs to store substantial amounts of carbon that contribute significantly to global carbon stocks.

## Data Availability Statement

All datasets generated for this study are included in the article/**Supplementary Material**.

## Author Contributions

VC, LC and MM conceptualized the research. GB-D-D, CE, IG-C and NS contributed to field sampling and data collection. VC conducted all soil analyses and estimations of soil carbon stocks. AL and LC-A conducted estimations of aboveground and belowground biomass and carbon stocks. LC-A, GB-D-D and LC performed all statistical analyses and drafted the manuscript. All authors reviewed and contributed to the manuscript.

## Funding

The study was supported through two grants from the Spanish Ministry of Economy and Competitiveness (CGL2013-45634-P, CGL2016-75414-P). GB-D-D was funded through a PhD grant by the Spanish Ministry of Education (FPU14/05303).

## Conflict of Interest

The authors declare that the research was conducted in the absence of any commercial or financial relationships that could be construed as a potential conflict of interest.
