# Supplementary material for: Trade-Offs Among Aboveground, Belowground, and Soil Organic Carbon Stocks Along Altitudinal Gradients in Andean Tropical Montane Forests
Source: Front Plant Sci. 2020 Mar 3;11:106. doi: 10.3389/fpls.2020.00106 (PMC7062916; doi:10.3389/fpls.2020.00106)
Supplement: Supplementary file 1 [file Presentation_1.zip › Supplementary material/Appendix S2.pdf]

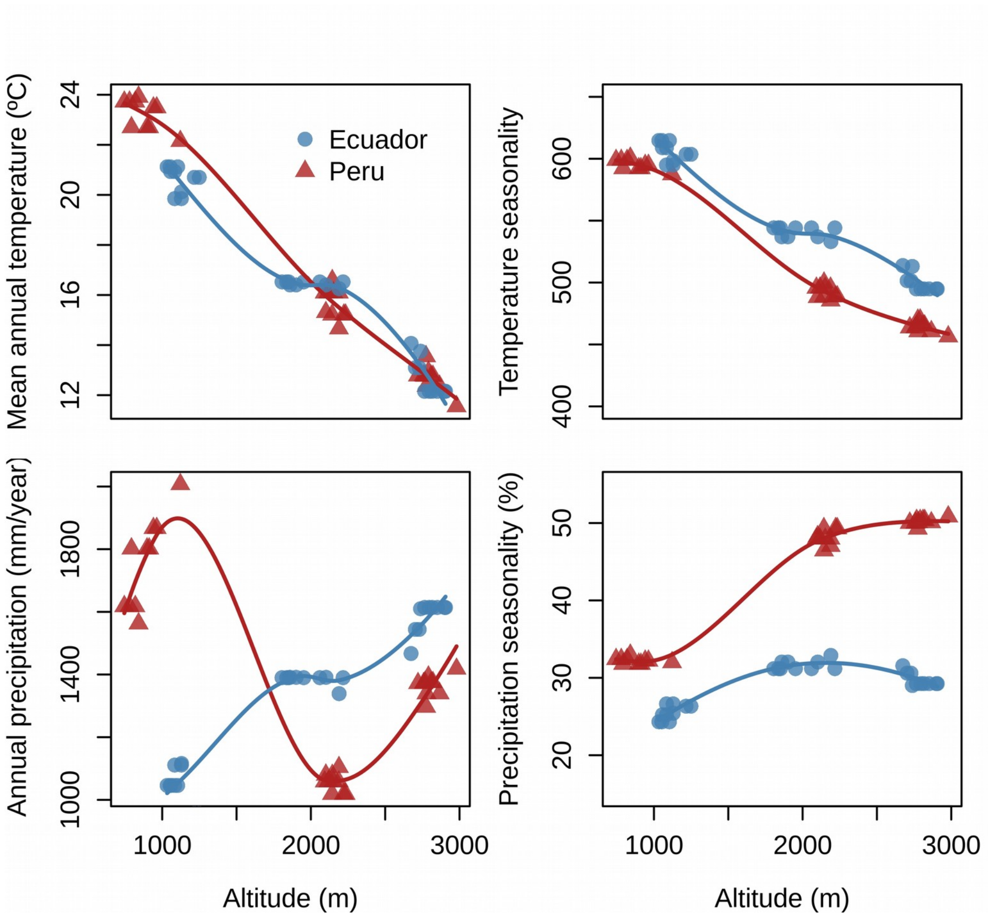

Appendix S2. Annual mean temperature (ºC), temperature seasonality, annual precipitation (mm/year), and precipitation seasonality (%) along altitudinal gradients in Ecuador and Peru sites. Local polynomial regression lines have been fitted to represent the relationship between the different climatic variables and altitude (m) at the two sites. Temperature seasonality is the standard deviation of monthly mean temperature; whereas precipitation seasonality is calculated as the standard deviation of the monthly precipitation estimates expressed as a percentage of the mean of those estimates (i.e. the annual mean) (Karger et al., 2017).
