# Supplementary material for: Trade-Offs Among Aboveground, Belowground, and Soil Organic Carbon Stocks Along Altitudinal Gradients in Andean Tropical Montane Forests
Source: Front Plant Sci. 2020 Mar 3;11:106. doi: 10.3389/fpls.2020.00106 (PMC7062916; doi:10.3389/fpls.2020.00106)
Supplement: Supplementary file 1 [file Presentation_1.zip › Supplementary material/Appendix S3.pdf]

Appendix S3. Summary of soil properties (pH, carbon, carbon/nitrogen ratio, bulk density, soil textures [percentage of sand, clay and silt] and coarse organic fraction weigth/total soil weight [COFW/TSW]) per altitudinal belt in Ecuador and Peru, where Low = 800-1100 m; Middle = 1900-2100 m; and High = 2700-2900 m.

**Altitudinal belt**	**pH**		**C (%)**		**C/N (%)**		**Bulk density (g cm^{-3})**		**Sand (%)**		**Clay (%)**		**Silt (%)**		**COFW/TSW (%)**	
	Ecuador	Peru	Ecuador	Peru	Ecuador	Peru	Ecuador	Peru	Ecuador	Peru	Ecuador	Peru	Ecuador	Peru	Ecuador	Peru
Low	4.82 ±0.75	5.46 ±1.13	10.4 ±6.8	5.7 ±1.3	14.1 ±1.2	11.1 ±0.8	0.76 ±0.16	0.65 ±0.15	73.2 ±5.2	52.4 ±6.4	15.3 ±5.2	19.6 ±3.6	11.5 ±4.18	28.0 ±6.56	4.0 ±4.7	0.6 ±0.3
Middle	4.09 ±0.56	5.48 ±0.45	17.2 ±12.8	16.4 ±9.0	16.8 ±2.4	13.9 ±1.6	0.53 ±0.09	0.43 ±0.18	65.0 ±9.2	70.4 ±2.0	17.5 ±4.0	13.7 ±0.5	17.5 ±6.6	15.9 ±2.0	1,0 ±0.6	2.1 ±1.5
High	3.28 ±0.75	4.41 ±0.30	32.4 ±17.4	20.1 ±6.4	22.9 ±8.3	15.5 ±1.6	0.43 ±0.07	0.29 ±0.13	72.6 ±1.5	82.9 ±0.7	16.2 ±1.1	11.9 ±0.1	16.2 ±1.1	5.2 ±0.6	2.1 ±1.1	9.4 ±12.4
