# Supplementary material for: Trade-Offs Among Aboveground, Belowground, and Soil Organic Carbon Stocks Along Altitudinal Gradients in Andean Tropical Montane Forests
Source: Front Plant Sci. 2020 Mar 3;11:106. doi: 10.3389/fpls.2020.00106 (PMC7062916; doi:10.3389/fpls.2020.00106)
Supplement: Supplementary file 1 [file Presentation_1.zip › Supplementary material/Appendix S4.pdf]

Appendix S4. Mean value and 95 % confidence intervals (in brackets) of the variables sampled at each altitudinal belt (diameter at breast height [DBH], tree height [H], wood density [WD]) per altitudinal belt in Ecuador and Peru, where Low = 800-1100 m; Middle = 1900-2100 m; and High = 2700-2900 m.

**Altitudinal Belt**	**DBH (cm)**		**H (m)**		**WD (g cm^3)**	
	Ecuador	Peru	Ecuador	Peru	Ecuador	Peru
Low	8.53 (2.60 – 34.10)	9.17 (2.50 – 41.73)	7.45 (2.80 – 18.00)	7.25 (2.00 – 20.00)	0.419 (0.161 – 0.704)	0.427 (0.167 – 0.685)
Middle	9.39 (2.80 – 36.42)	8.34 (2.50 – 36.22)	8.09 (3.00 – 20.00)	5.70 (2.00 – 15.00)	0.415 (0.173 – 0.631)	0.377 (0.141 – 0.616)
High	6.61 (2.80 – 18.30)	8.30 (2.50 – 34.75)	5.62 (3.00 – 9.50)	6.02 (2.00 – 16.00)	0.463 (0.244 – 0.648)	0.352 (0.108 – 0.601)
